# Current News and Opinion

**Published:** 1883-07

**Authors:** 


### Current News and Opinion.

### GOOD ADVICE TO TRAVELERS.

Dr. C. W. Chancellor, in a recent letter from Geneva to the *Baltimore Day*, gives the following excellent advice to European trav_ elers :

"I feel I would be but ill acquitting myself of a duty were I to fail to administer an admonition to those of my compatriots who may one day journey into this land, and I hope they will take heed to what I say, for it is wholesome. I would strongly advise Americans who contemplate traveling upon the continent to be very chary of patronizing physicians recommended by hotel or boarding-house keepers, concierges, porters, etc., etc., without first having inquired of their consul or their banker, or some friend as to the standing of the party recommended, for it not infrequently happens that these parties plot together exclusively as a matter of personal gain, and without any regard whatever for the well-being or interest of those whom they advise.

"It would be well for persons visiting Europe either to obtain the addresses of competent medical men in the various cities they propose visiting before leaving home, or on their arrival to get advice from some reputable person out of business and above taking a commission, otherwise they may have a tenth-rate doctor introduced as the 'former physician to the Emperor,' the 'chief of the hospitals,' the 'doctor of the American Legation,' or some other high-sounding but fictitious title, and they may be left in his hands to be robbed, maltreated, and perhaps murdered.

"Travelers, in fact, should make it a rule to take any other physician than the one proposed by a landlord or concierge or courier, unless the medical man thus recommended be a compatriot, or is endorsed by some disinterested person; and they should insist upon having the doctor of their choice—if they have a choice—really sent for, taking no excuse for any delay or neglect in regard to the matter.

"There are reliable and veritable American physicians in nearly all of the large cities of Europe whose addresses can readily be found by consulting the *Directory*, which is in the office of every respectable hotel, or by inquiring at the nearest drug-store."

[This advice is as applicable in the selection of a dentist when his services are needed, as in the choice of a physician.—EDITOR.]

---

### NEW JERSEY STATE DENTAL SOCIETY.

The thirteenth annual meeting of this Society will be held at the Coleman House, Asbury Park, commencing Wednesday, July 18 1883, at ten o'clock, and will continue in session until adjournment.

### PROGRAMME.

1.—Annual Address by the President, Dr. J. G. Palmer, New Brunswick.

2.—The Different Preparations of Gold for Filling Teeth. By Dr. George C. Brown, Elizabeth.

3.—Æsthetic Dentistry. By Dr. E. Harvey Bunting, Newark.

4.—The Legal and Moral Responsibility of Dentists in the Administration of Nitrous Oxide Gas. By Dr. J. Allen Osmun, Newark.

5.—Lobelia Inflata, as a Therapeutic Remedy. By Dr. J. W. Scarborough, Lambertville.

6.—Filling Teeth, and the Philosophy thereof. By Dr. J. Hayhurst, Lambertville.

7.—Volunteer Essay. By C. W. Meloney, Camden.

8.—Volunteer Essay. By Dr. Harvey Iradell, New Brunswick.

### CLINICS.

Dr. C. A. Timmie, Hoboken. Exhibition Hydraulic Compressor for Swaging Perfect Metal Plates in five minutes.

The Berlin Continuous Gum Furnace for Baking Continuous Gum Sets in thirty minutes.

Dr. C. F. W. Bodecker, New York. Stereopticon Exhibition on Screen, Descriptive of the Anatomy and Pathology of Tooth Tissue.

Dr. E. Slegel, Reading. New Method of Inserting Crowns.

Dr. F. W. Buttner, New York. Dr. Buttner's Artificial Crown.

Dr. W. C. Gardinere, Marseilles, France. New Cast Metal Base.

### THE AMERICAN DENTAL ASSOCIATION.

The twenty-third annual meeting of this Association, which meets at Niagara Falls, on Tuesday, August 7th, will be held in one of the parlors of the International Hotel. The "Pavilion" in the Park, could not be secured for the purpose, and the committee having the matter in charge has thought this preferable to again renting the unsatisfactory Grant's Hall. Reduced rates have been secured at both the International and Cataract Houses, as well as at all places of interest where an entrance fee is charged. All the principal eastern and western railways will grant round trip tickets for one fare and a third, but to secure this reduction of rates an application for a certificate of membership must be secured from Dr. Geo. L. Field, Detroit, Mich. Those wishing such certificates should make application as early as possible.

Geo. L. Field,<br>

F. M. Odell, } *Committee.*<br>

T, T. Moore,

---

### AMERICAN SOCIETY OF MICROSCOPISTS.

It is unfortunate that the sixth annual meeting of this Society was called for the same week in which occurs the meeting of the American Dental Association, as this will deprive a number of members of the privilege of attendance. The meeting will be held in Chicago, commencing Tuesday, August 7th, and will continue four days. The President is Prof. Albert McCalla, of Fairfield, Iowa. The Secretary is Prof. D. S. Kellicott, Buffalo, N. Y.

---

### AMERICAN DENTAL CONVENTION.

This Society will hold its next annual meeting at Saratoga Springs, on the second Tuesday of August, 1883. A large attendance is expected, and a number of interesting papers are already promised.

A. C. Rich, *Secretary.*<br>

Saratoga, N. Y.

### NOTICE TO STATE BOARDS OF EXAMINERS.

There will be held at the Cataract House, Niagara Falls, on Monday, August 6th, 1883, at 2 o'clock P. M., a meeting of all the State Boards of Dental Examiners, for the purpose of perfecting the organization of a National Association of Examining Boards. It is hoped that every Board will be fully represented.

GEORGE H. CUSHING,
*Secretary of Conference held at Lexington, Ky.*

---

### MAINE DENTAL SOCIETY.

The Maine Dental Society will hold its eighteenth annual meeting in Portland, on Tuesday and Wednesday, July 17 and 18, 1883.

D. W. FELLOWS, *Secretary.*

